# Temperature, size and developmental plasticity in birds

**DOI:** 10.1098/rsbl.2022.0357

**Published:** 2022-12-07

**Authors:** Brian C. Weeks, Madeleine Klemz, Haruka Wada, Rachel Darling, Tiffany Dias, Bruce K. O'Brien, Charlotte M. Probst, Mingyu Zhang, Marketa Zimova

**Affiliations:** ^1^ School for Environment and Sustainability, University of Michigan, Dana Building, 440 Church Street, Ann Arbor, MI 48109, USA; ^2^ Department of Biological Sciences, Auburn University, Auburn, AL, USA; ^3^ Department of Biology, Appalachian State University, Boone, NC, USA

**Keywords:** developmental plasticity, endotherms, Bergmann's rule, body size, climate change, phenotypic plasticity

## Abstract

As temperatures increase, there is growing evidence that species across much of the tree of life are getting smaller. These climate change-driven size reductions are often interpreted as a temporal analogue of the observation that individuals within a species tend to be smaller in the warmer parts of the species' range. For ectotherms, there has been a broad effort to understand the role of developmental plasticity in temperature–size relationships, but in endotherms, this mechanism has received relatively little attention in favour of selection-based explanations. We review the evidence for a role of developmental plasticity in warming-driven size reductions in birds and highlight insulin-like growth factors as a potential mechanism underlying plastic responses to temperature in endotherms. We find that, as with ectotherms, changes in temperature during development can result in shifts in body size in birds, with size reductions associated with warmer temperatures being the most frequent association. This suggests developmental plasticity may be an important, but largely overlooked, mechanism underlying warming-driven size reductions in endotherms. Plasticity and natural selection have very different constraining forces, thus understanding the mechanism linking temperature and body size in endotherms has broad implications for predicting future impacts of climate change on biodiversity.

## 1. Introduction

Across the diversity of life, there is a tendency for individuals in the warmer parts of a species' range to be smaller. This spatial pattern—an intraspecific derivative of Bergmann's rule [1–3]—has led to predictions of similarly general reductions in size as the world warms [4,5]. Despite some variation in temperature responses among species [5], evidence of warming-driven size reductions is accumulating both across distantly related taxonomic groups [4,6], and within groups of both endotherms [7–10] and ectotherms [6,11,12]. Body size influences nearly all aspects of an organism's life history [13,14], including physiological tolerances, reproductive capacity and interspecific interactions, and is a key determinant of the contributions of individuals to ecosystem functioning (the stocks, or fluxes, of energy and materials in a natural system; [15,16]). Should widespread reductions in body size in response to warming temperatures be maladaptive, they have the capacity to reduce individual and population fitness [17,18] with broad implications for ecosystems [15,16]. Despite growing evidence of climate change-driven size reductions, there is considerably less known about the processes linking temperature and size [19].

The two main hypothesized explanations for observed relationships between size and temperature are natural selection and phenotypic plasticity. The original explanation for why organisms tend to be larger in colder regions is that the lower ratio of surface area to volume for larger individuals confers a selective advantage in colder climates by reducing convective heat loss [1,2,20,21]. Building on this idea, in the context of global warming, natural selection for smaller body size to increase convective heat loss in warmer climates is a commonly invoked mechanism for the observed warming-associated reductions in body size [22]. However, the relationship between temperature, heat dissipation and body size is complex. For example, water requirements for evaporative heat loss are greater under increasing temperatures and this higher demand for water is mass-dependent, with smaller birds experiencing greater increases in water demand [23]. Thus, either relaxed selection for cold tolerance or increased selection for more effective heat dissipation might connect warming temperatures and size reductions, but clearly this relationship is expected to be complex.

While selection is one potential mechanism underlying observed linkages between temperature and body size [4,7,8], it is also possible that warming-driven size reductions are the result of changes to the conditions experienced during development that alter the expression of the underlying genotype (i.e. phenotypic plasticity) [24]. In ectotherms, temperature-mediated developmental plasticity is thought to be a widespread mechanism linking higher temperatures during development with smaller body size at maturity, a pattern known as the temperature–size rule (TSR; [5,25]). The rates of many physiological processes increase approximately exponentially with temperature [26,27]. Warmer environmental temperatures increase metabolic rates and rates of cellular processes in ectotherms, resulting in faster growth and development and smaller body size at maturity [28–30]. This ‘pace of life’ explanation for the TSR, in which more rapid growth is correlated with smaller size at maturity, is not the only mechanism that has been put forward to explain the connection between temperature and size in ectotherms (notably, reduced oxygen availability is a dominant explanation of the TSR; [31–35]), however, the bulk of the hypothesized mechanisms underlying the TSR in ectotherms invoke developmental plasticity rather than selection [36].

By contrast to ectotherms, in birds and mammals, temperature-mediated developmental plasticity has just recently been invoked as a broadly important mechanism linking warming and observed reductions in body size [24]. This is potentially attributable to the ability of endotherms to buffer their metabolic processes from shifts in ambient temperatures by actively maintaining their body temperatures [37], making them appear less likely than ectotherms to experience variation in physiological processes as a result of differences in environmental temperatures. In fact, there is a long history of empirical work in poultry science demonstrating a permanent change in physiological function and body size due to developmental temperature (see below). Although this literature is rarely considered in wildlife studies, it suggests that developmental plasticity—particularly at the embryonic stage—has the potential to play a major role in observed warming-driven size reductions in natural systems [24,38]. The mechanism underlying temperature-associated differences in size has important implications for understanding and predicting the fitness consequences of climate change.

## 2. Evidence of temperature-driven developmental plasticity in birds

### (a) Phenotypic plasticity in endotherms related to size

In natural settings, while adult endotherms may be able to buffer themselves from variation in environmental temperatures via thermoregulation, some young birds (e.g. altricial birds like passerines) and young mammals have little ability to thermoregulate and are effectively poikilothermic (i.e. they are unable to regulate their body temperature other than through behaviour; [39–44]). Therefore, at critical periods during development, exposure to increased temperatures may induce developmental plasticity. Additionally, changes in resource availability driven by shifting abiotic conditions can induce phenotypic plasticity through altered resource availability [45], providing an indirect link between temperature and size.

### (b) A review of the experimental evidence of temperature-driven developmental plasticity in birds

We review over half a century of relevant work in both laboratory and natural settings to determine the role of temperature-mediated developmental plasticity in warming-driven size reductions in birds, and compare experimental and observational results. Results from experiments that focus on within-generational impacts of temperature treatments on size and development cannot be explained by selection; rather, they reflect physiological and morphological responses to temperature by the individual. Should developmental plasticity be a significant mechanism underlying warming-driven size reductions in endotherms, it would have important implications for future morphological consequences of climate change in birds, and could explain both the variation in size responses to temperature within taxonomic groups [4] and consistent changes in size across ecologically distinct species [8].

We conducted a systematic search of the literature, using keyword co-occurrence networks and text-mining [46] to identify over 2000 potentially relevant studies (see the electronic supplementary material (ESM) for methodological details). We reviewed the titles and abstracts of these papers and ultimately identified 178 studies published from 1972 to 2020 that examined the relationship between temperature and avian development for 106 wild bird species. These were composed of 90 studies based on observational data and 91 studies that manipulated ambient temperature and measured the effects of temperature on physiology or morphology.

We focus on 51 of these studies that assessed the relationship between temperature and size for 31 different species (ESM, table S1). Of these, 34 experimentally manipulated the temperatures of eggs or nestlings (individuals after hatching and pre-fledging) and 18 included observational data (one study included both experimental and observational data). Of the relationships between temperature and size reported in the manipulative studies, 64% reported a significant relationship between temperature and size, with the most common response to both heating and cooling treatments being a reduction in size (figure 2*a*; ESM). Within the studies that experimentally increased ambient temperature, 61% of the relationships recovered were a decline in size, 4% were an increase in size and 36% were not significant (figure 2*a*). Some of the studies that found warming-driven size reductions found that the warmer temperatures were also associated with faster growth rates [47–52]. This suggests a mechanism similar to the ‘pace of life’ explanation in ectotherms, although this pattern was not universal [53–55].

**Figure 1.**
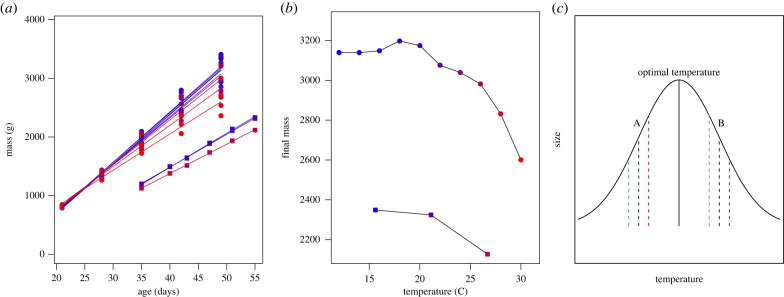
(*a*,*b*) Warmer temperatures during development lead to smaller size. Across two studies (circles [57], and squares [58]), increasing temperatures (redder lines) resulted in reduced size (*a*). The relationship between the final size and temperature (*b*) is nonlinear; at low temperatures, increasing the temperature during development either does not change or slightly increases size, while at higher temperatures, increases in temperature drive a reduction in size. This suggests a general negative relationship between temperature and size, but with the added complexity that temperature effects are variable across rearing temperatures. (*c*) The impacts of warming or cooling on developmental plasticity may be determined by the relationship between ambient and optimal developmental temperatures. If the ambient temperature, A, is below the optimal developmental temperature for a species, warmer temperatures during development may result in an increase in size (red line), and cooling may reduce size (blue line). Conversely, if ambient temperature, B, is at or above optimal, increasing temperatures may reduce size (red line) while cooling may increase size (blue line). Optimal developmental temperatures and the form of this relationship are unknown for the vast majority of endotherms.

**Figure 2.**
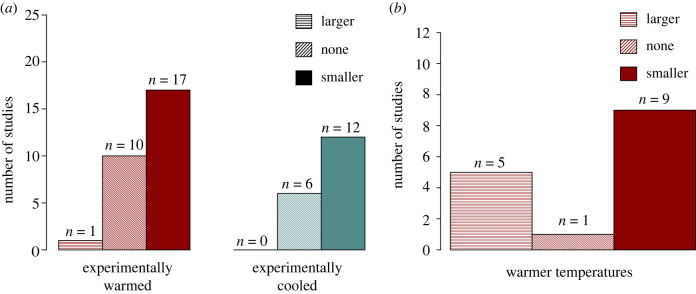
(*a*) Plastic changes in size were observed in response to both experimental warming and cooling. For those studies that experimentally tested size responses to warming, 61% of the relationships recovered were warming-driven declines in size. While fewer studies experimentally cooled nests, there was a high rate of cooling-driven size reductions (67%). (*b*) As with the experimental results, observational studies report a widespread relationship between warmer temperatures and size, with reductions in size occurring in a majority (60%) of instances. These findings are robust to the inclusion of only a single study per species in each category (ESM).

While warming-driven size reductions were the most frequently reported outcome, there were other temperature–size relationships. Although studies that experimentally decreased rearing temperature (*n* = 15) were less common than warming experiments (*n* = 26), a higher proportion (67%) of the relationships recovered in the cooling studies were declines in size. And—while it was less common than size reductions—4% of the warming treatments resulted in larger size (figure 2*a*). It is possible that whether higher temperatures result in an increase or decrease in size may depend on where ambient temperatures stand in relation to the optimal temperature for development (used here to mean the temperature that results in the largest offspring) for a species [56]. If ambient temperatures are at, or above, the optimum temperature for development, higher temperature may result in smaller size; conversely, if ambient temperatures are below the optimum temperature, higher temperature may result in larger size as it approaches an optimum temperature (figure 1*c*). In addition to data that are consistent with this hypothesis from poultry, there are several examples of studies that warmed nests of non-model species during the nestling phase and found results that follow this pattern. A mild increase of nest temperature to 31.8°C produced heavier nestlings in tree swallows [59] while heating nests to 44.1°C, with maximum temperatures above 50°C, well above the thermoneutral temperature range of blue tit nestlings (35–40°C; [60]) suppressed nestling growth of blue tits [61]. Importantly this phenomenon may vary across life stages. For example, the relationship between the experimental incubation temperature relative to the optimal incubation temperature and its effect on body size and growth may be different during the incubation phase when lower than optimal incubation temperature may reduce efficiency of yolk usage leading to consistently low growth for high yolk usage [62], with less consistent effects after hatching [63,64]. Understanding the extent to which a nonlinear relationship between temperature and size may explain these apparently conflicting findings will require a broader knowledge of the optimal temperatures for development across species. Comparative studies may also reveal important roles of life-history strategy and species-specific physiology in mediating temperature–development relationships. Further, smaller body size may not be maladaptive; understanding how differences in body size relate to fitness across a range of environmental conditions is thus critical to interpreting the consequences of temperature–size relationships.

### (c) Interpreting observational results in light of the experimental findings on temperature-mediated plasticity

While experimental manipulations are necessary to isolate the effects of developmental plasticity from potentially confounding impacts of natural selection, it is possible to qualitatively compare temperature–size relationships in experimental and observational studies using the studies we review. Eighteen of the observational studies we reviewed quantified the relationship between temperature and size (figure 2*b*), and 14 of these (78%) were exploring the impacts of warmer temperatures on size. Of the relationships recovered in these studies, 60% found that warmer temperatures were associated with reduced body size, 33% found that warmer temperatures were associated with larger body size and 7% reported no relationship.

If both selection and plasticity were contributing to observed warming-driven size reductions, we might expect a higher proportion of the observational studies to find warming-driven size reductions. This assumes additionality, in which both developmental plasticity and selection would contribute to size reductions, as opposed to only developmental plasticity in the experimental studies. However, we do not find evidence of this. Roughly the same proportion of relationships in the correlational (60%) and experimental (61%) studies find warmer temperatures are associated with size reductions, and a higher proportion of the correlational warming studies reported an increase in size (33%) than in experimental studies (4%). Our results are thus consistent with a relatively important role for developmental plasticity in driving warming-driven size reductions in the observational studies, although it should be noted that the limited sample size, relatively subtle variation in temperature in the observational studies and potential for confounding processes that covary with temperature are important caveats to this interpretation.

## 3. Potential mechanistic drivers of temperature-mediated developmental plasticity in endotherms

### (a) Resource-driven phenotypic plasticity

One possible pathway by which warming might induce phenotypic plasticity-driven size reductions is through temperature-related changes in resource availability. Unlike ectotherms, the young of many birds and mammals rely solely on food provided by their parents. For birds, when ambient temperature increases, provisioning rates and total prey delivered by the parents can decrease, resulting in smaller and lighter fledglings [65–67]. Similarly, in mammals lactation can be limited by higher temperatures as a result of constraints on energy expenditure imposed by heat dissipation capacity [22], and this can ultimately reduce offspring size [68]. Consistent with this mechanism, in mammals, the impacts of temperature on size are mediated by resource availability and life-history strategies [69]. Thus, temperature effects on phenotypic plasticity driven by changes in resource availability may act in combination with direct effects of temperature on developmental plasticity. However, consistent declines in size associated with warming temperatures across diverse groups of endothermic species [8], and warming-driven reductions in size when feeding rates are kept consistent in experimental settings [70], suggest a role for a fundamental physiological link between ambient temperature and size. While resource availability could impact the observational studies we review, the plastic responses to nest warming experiments are unlikely to result from changes in resource availability, as the ambient temperatures and thus temperature-mediated resource availability remained unchanged.

### (b) Heat stress, developmental plasticity in the IGF pathway and body size

In addition to plasticity resulting from the effects of temperature on resource availability, temperature may directly impact key physiological determinants of body size, such as growth hormones and insulin-like growth factors (IGFs). In particular, the relationship between IGFs and size is well known, yet is rarely considered as a potential mechanism in studies investigating climate-change driven size reductions. IGF signalling pathways regulate cellular metabolism, proliferation and differentiation and are thought to mediate the effects of environmental conditions on growth, body size, fecundity and senescence [71,72]. Broadly across passerines, there is a positive relationship between IGF levels and mass [73], and experimental increases in IGFs during embryonic and fetal development result in increased gestational and postnatal body size and mass in birds and mammals [74–76]. Temperature during development can impact IGF levels directly (i.e. this effect is not mediated through temperature effects on resource availability), with higher temperatures associated with reductions in serum IGF levels and gene expression of IGFs [70]. Such a direct relationship between temperature and IGF levels suggests heat-driven plasticity in IGF levels is a potential mechanistic link between increasing temperatures and size reductions in birds.

### (c) Temperature, developmental plasticity and IGFs in model systems

The production of large individuals has long been a primary goal of poultry science, and as a result the relationship between temperature and size has been thoroughly characterized in chickens (*Gallus gallus domesticus*). There is a rich history of replicated experiments in which large numbers of genetically similar chicks are reared under controlled conditions, making it possible to isolate the effects of temperature on development. While there is significant variation in the methodologies of these papers, including the degree, duration and life stage of experimental manipulation, key generalities have emerged. In particular, warmer temperatures tend to result in smaller individuals. For example, two studies with carefully standardized methodologies and high sample sizes (*n* = 4240) that reared chickens across a range of temperatures [57,58] found a general relationship between increasing temperatures and smaller body size (figure 1*a*). However, this relationship is nonlinear. At low temperatures, there is no reduction, or even a slight increase, in size as temperature increases; this is followed by a negative relationship between temperature and size at higher temperatures (figure 1*b*). It is possible that developmental plasticity in the IGF pathways may underlie this connection between temperature and size.

The impacts of temperature on IGF pathways are complex. In particular, the duration, timing and severity of the manipulation of temperature during development determines whether the thermal stress increases [77,78] or decreases [64,79–81] size. In chickens, short, intermittent exposure to heat during mid- or late-incubation can improve post-hatch growth rate and is associated with elevated IGF expression during the treatment and post-hatch development [82,83]. On the other hand, prolonged heat exposure can lead to reductions in body mass and body size, with high ambient temperatures reducing body mass independently of any effect on food intake [70]. Ma *et al*. [70] showed that developing chickens that were exposed to heat stress (ambient temperatures were held at 32°C for 14 days) had lower body mass gain compared to non-heat stressed chickens (reared at 22°C), even when the non-heat stressed group was pair-fed (i.e. its food intake was constrained to be the same as the food intake of the heat-stressed group). Serum IGF and IGF receptor expression in the breast muscle were both significantly lower in the heat stress group compared to the non-heat stressed group. Observed reductions in receptor expression likely reduce the efficacy of IGF, as a study with mutant newborn mice lacking IGF receptors showed that those mice suffered several developmental delays including delays in bone development of 1 to 2 days, as well as reduced number of myocytes and dwarfism [84]. IGF1 binding to IGF1 receptors activates the IGF1/protein kinase B pathway which in turn promotes protein synthesis and thus muscle growth through mammalian target of rapamycin (mTOR) and 70 kD ribosomal protein S6 kinase (S6K1) [85]. Interestingly, chronic heat stress in broiler chickens reduced both mTOR and S6K1 gene expression in breast muscles [70] indicating that heat stress reduces body mass by downregulating the IGF1/protein kinase B pathway, independently from temperature impacts on food intake.

The relationship between IGF and size is not limited to birds. Transgenic mice embryos with a local elevation of IGF gene expression in skeletal muscles had increased muscle mass and fibre area [86], and reduced serum IGF levels in dogs are associated with smaller size [87]. Given the suppression of IGF pathways that results from chronic heat stress in birds, and the broad correlation between IGF levels and size in birds and mammals, temperature impacts on the IGF axis are one possible physiological mechanism underlying developmentally plastic size reductions associated with global warming.

To date, experimental work on the effects of temperature manipulations on the IGF axis, particularly in the context of climate change, has been limited. Given the potential significance of this relationship, work testing whether IGF pathways link climate change and size reductions across diverse sets of species is an exciting avenue of future research. Further, very little is known about the impacts of sub-optimally cold temperatures on the IGF pathway, thus the ability of IGF pathways to explain the connections between reduced temperatures and smaller body size (figure 2*a*) remains an open question.

### (d) Other possible physiological mechanisms

Aside from the potential direct effect of heat on IGFs, other neuroendocrine mechanisms, such as glucocorticoids and thyroid hormones, may also play a role in mediating heat-associated size reduction in endotherms. High ambient temperatures can stimulate the hypothalamic–pituitary–adrenal axis and elevate glucocorticoid secretion from the adrenals [88]. Chronic and excessive elevation of glucocorticoids reduces skeletal growth both directly, by increasing bone resorption and inhibiting osteoblast activity, and indirectly through interfering with the growth hormone/IGF axis [89–91]. Taken together, this suggests glucocorticoids are likely to facilitate heat-associated suppression in skeletal growth.

Thyroid hormones are essential for normal development and play a key role in thermoregulation and metabolism [92]. For instance, hypothyroidism during postnatal development can halt growth in humans if untreated [89]. In birds, embryonic and postnatal exposure to heat has mixed results on thyroid hormone levels [93], thus, whether high ambient temperature can influence body size through the hypothalamic–pituitary–thyroid axis warrants further investigation.

It is also possible that heat-associated reductions in body size are due to reduced feed efficiency (i.e. the efficiency of conversion of feed into tissue). Altricial birds, such as passerines, cannot thermoregulate for 1 to 2 weeks in the nestling stage [94], and while little is known about the relationship between ambient temperature and feed efficiency in young birds, some ectotherms (e.g. fish) show reduced feed efficiency at low and high temperature during development [95,96].

## 4. Limitations and outstanding questions

While there is a long history of experimental work linking temperature during ontogenetic growth and development, much is left to be learned. Across the studies we have synthesized, there was large variation in the temperature treatments; some studies relied on extremely coarse warming measures (e.g. putting candles underneath nests [97]) and there was a wide range in the intensity—both the duration and degree of temperature change—of the temperature treatments, which may be important determinants of the temperature effects. Further, across the studies there was variation in the developmental stage subjected to the temperature manipulations, and different indicators of size effects (e.g. changes in mass, or the length of the tarsus or wing) were measured at various life stages (ESM, table S1). While this lack of standardization precluded making more detailed consensus statements and may have reduced the consistency within our results, there is no reason to expect that the noise added to the data as a result of this complexity would alter the direction of the patterns we recover. Additional work on this topic may lead to insights into study- and species-specific attributes that determine the magnitude and consistency of temperature–development relationships. While birds and mammals are both endotherms, and many of their developmental responses to thermal pressures are similar [98], they have myriad differences in their physiology and reproductive biology that likely result in different impacts of thermal conditions on development. For example, external development in birds likely exposes bird embryos to greater fluctuations in temperature than embryos of non-monotreme mammals [98]. There remain important outstanding areas of uncertainty surrounding the impacts of thermal changes on development. These are particularly pronounced in mammals for which data are limited compared to birds [98], but in birds the relationship between warming temperatures and development is sensitive to a range of variables including the time in development of warming (e.g. pre- or post-hatching) [24], the reproductive strategy of birds [98] and how increasing climate variability may influence temperature-development relationships in birds [98], and these remain active areas of research.

It is also possible that the results of warming experiments may not be accurate indicators of the role of phenotypic plasticity in natural systems. Parental behavioural change in response to warmer ambient temperatures could dampen or strengthen phenotypic plasticity in offspring. Parents can actively select nest locations to take advantage of cooler microclimates [99], potentially mitigating the effects of increasing temperatures on offspring. Conversely, higher temperatures can cause individuals to change foraging behaviour, reducing foraging efficiency [100], and even leading to negative growth on particularly hot days [101], and alterations in parental behaviour as temperatures warm may further expose pre-hatching offspring to thermal stress [102]; broadly, behavioural changes in response to temperature can incur costs—including reduced foraging, offspring care and defence of territories [103]—and these costs may be important determinants of vulnerability of species to climate change [103]. Clearly, developmental plasticity is only part of a complex suite of factors guiding phenotypic plasticity. Given that many of the experiments we reviewed did not integrate parental behaviour in a warmer world, the influence of parental responses to climate on offspring development might reduce or exacerbate the relationships we find. As such, it is possible that parental behaviour might shield young from experiencing extreme temperature and reduce the contribution of developmental plasticity to warming-driven size reductions in the wild. Additionally, there is a bias toward temperate species and systems in the data, with limited analyses of tropical species outside of Australia; this bias exists in observational studies as well, and highlights a priority for future work.

Finally, it is possible that publication bias—in particular, lower rates of publishing non-significant effects of temperature treatments—may have inflated the percentage of studies in which responses to warming or cooling treatments occurred. While this has the potential to have inflated the percentage of studies that find warming-driven size reductions, our conclusion that developmental plasticity has the capacity to link temperature and size in birds is robust to this potential complication.

## 5. Conclusion

Widespread shifts in body size across diverse groups of species have the potential to have far-reaching consequences for the persistence of individual species and the functioning of natural systems [5,15]. Despite evidence of warming-driven body size reductions in both ectotherms and endotherms, the attribution of this trend to developmental plasticity has largely been confined to ectotherms. However, we find that there is a large body of empirical evidence which suggests a role for developmental plasticity in warming-driven size reductions in birds, consistent with previous synthetic [19,24,104] and single-species [38] efforts to understand the mechanistic link between variation in air temperature and size. Additional comparative research is needed to tease apart general processes and species-specific attributes that modulate the temperature–development relationship. Drawing on the extensive empirical research in poultry science, we propose that temperature effects may be impacted by the relationship between ambient and optimum temperatures for development. Finally, we synthesize evidence of the effects of temperature on IGF levels and hypothesize that IGF pathways may underlie this phenomenon.

The expression of different phenotypes depending on environmental conditions during development can result in permanent physiological or behavioural changes. Should this developmental plasticity shift traits to better match the demands of environmental conditions, they may improve fitness [105,106]; however, data on the fitness benefits of developmental plasticity driven by thermal conditions is limited [93]. There is some evidence that individuals that are more sensitive to developmental temperature have greater post-fledging survival, suggesting plasticity can improve fitness [56,107]. However, plasticity is not always adaptive. When the environmental cues that drive developmental plasticity are unreliable indicators of trait optima, plasticity can lead to population declines [108]. Further, changes in the thermal environment can impact aspects of the phenotype beyond size, including thermoregulatory control, which can be constrained or enhanced by variation in developmental temperatures [98], and warmer temperatures can have direct deleterious effects on the physiology of developing individuals [109]. Even when plasticity might be adaptive, it may be insufficient to prevent long-term fitness declines [110], and there is little evidence of selection for plasticity in response to thermal conditions [111]. Thus, even if plastic responses to rising temperatures are currently conferring fitness benefits, the potential for plasticity to mitigate the impacts of continued global warming may be constrained, though the fitness consequences of temperature-dependent development remains an open question.

Natural selection and plasticity are non-exclusive potential mechanisms of temperature-driven changes in body size. While natural selection can drive directional changes in the phenotype, evolutionary changes are slow relative to plastic changes [21,112] and may be less uniform across species than plasticity operating on essential physiological processes. Understanding the relative importance of natural selection and plasticity in mediating the relationship between temperature and morphology in endotherms should be a priority. As the world continues to warm, these mechanisms have different implications for both the universality, fitness consequences and future trajectories of warming-driven morphological change.

## Data Availability

The data are provided in electronic supplementary material [113].
